# Acupuncture promotes muscle cells ATP metabolism in ST36 acupoint local exerting effect by activating TRPV1/CaMKII/AMPK/PGC1α signaling pathway

**DOI:** 10.1186/s13020-025-01169-z

**Published:** 2025-07-14

**Authors:** Zhihan Chen, Kaifang Yao, Xinrui Wang, Yangyang Liu, Simin Du, Shenjun Wang, Yuxin Fang, Yuan Xu, Zhifang Xu, Xiaowei Lin, Yi Guo

**Affiliations:** 1 School of Acupuncture & Moxibustion and Tuina, Tianjin University of Traditional Chinese Medicine, Tianjin, 301617 People’s Republic of China; 2 Research Center of Experimental Acupuncture Science, Tianjin University of Traditional Chinese Medicine, Tianjin, 301617 People’s Republic of China; 3 School of Traditional Chinese Medicine, Tianjin University of Traditional Chinese Medicine, Tianjin, 301617 People’s Republic of China; 4 Tianjin Key Laboratory of Modern Chinese Medicine Theory of Innovation and Application, Tianjin, 301617 People’s Republic of China; 5 National Clinical Research Center for Chinese Medicine Acupuncture and Moxibustion, Tianjin, 300381 People’s Republic of China; 6 State Key Laboratory of Component-Based Chinese Medicine, Tianjin University of Traditional Chinese Medicine, Tianjin, 301617 China

**Keywords:** Acupuncture, ST36,TRPV1, ATP

## Abstract

At present, a number of studies have shown that acupuncture at Zusanli (ST 36) can relieve pain, but the changes of local microenvironment in the acupoint area after acupuncture have not been elucidated. As a temperature and pain receptor, TRPV1 plays an important role in pain perception and inflammation regulation. In this study, RT-PCR technique was used to screen the types of mechanically sensitive ion channels in the local response to acupuncture in the acupoint area, and western bolt technique was used to verify in gene knockout and antagonist injection mice. Immunofluorescence double labeling technique was used to further determine the key cell types of TRPV1-mediated acupuncture analgesia. Finally, through the combined analysis of proteomics and phosphorylated proteomics, the local signaling pathways of acupoints that can be activated by acupuncture were analyzed. This study systematically explored the analgesic effect of acupuncture on inflammatory pain in mice and its mechanism. The study found that acupuncture can significantly improve the thermal pain threshold and mechanical pain threshold in mice, showing a significant analgesic effect. Further analysis revealed that this analgesic effect was closely related to the up-regulation of local TRPV1 expression at ST36, and its deletion or functional inhibition would significantly weaken the analgesic effect of acupuncture. In addition, we also found that acupuncture in the deep muscle layer can more effectively promote the expression and activity of TRPV1 than in the superficial fascia layer, and muscle cells are the key cell types of TRPV1-mediated acupuncture analgesia. Finally, through the combined analysis of multi-omics, it was clear that acupuncture could activate the local signal pathway TRPV1/CaMKII/AMPK/PGC1α to exert analgesic effect. In conclusion, this study not only confirmed the analgesic effect of acupuncture on inflammatory pain in mice, but also revealed the core role of TRPV1 in the mechanism of acupuncture analgesia, especially the important contribution of TRPV1 expression and activity in the muscle layer of ST36 acupoint to the analgesic effect of acupuncture, which provided a new scientific basis and potential therapeutic target for acupuncture treatment of inflammatory pain.

**Graphical abstract:**

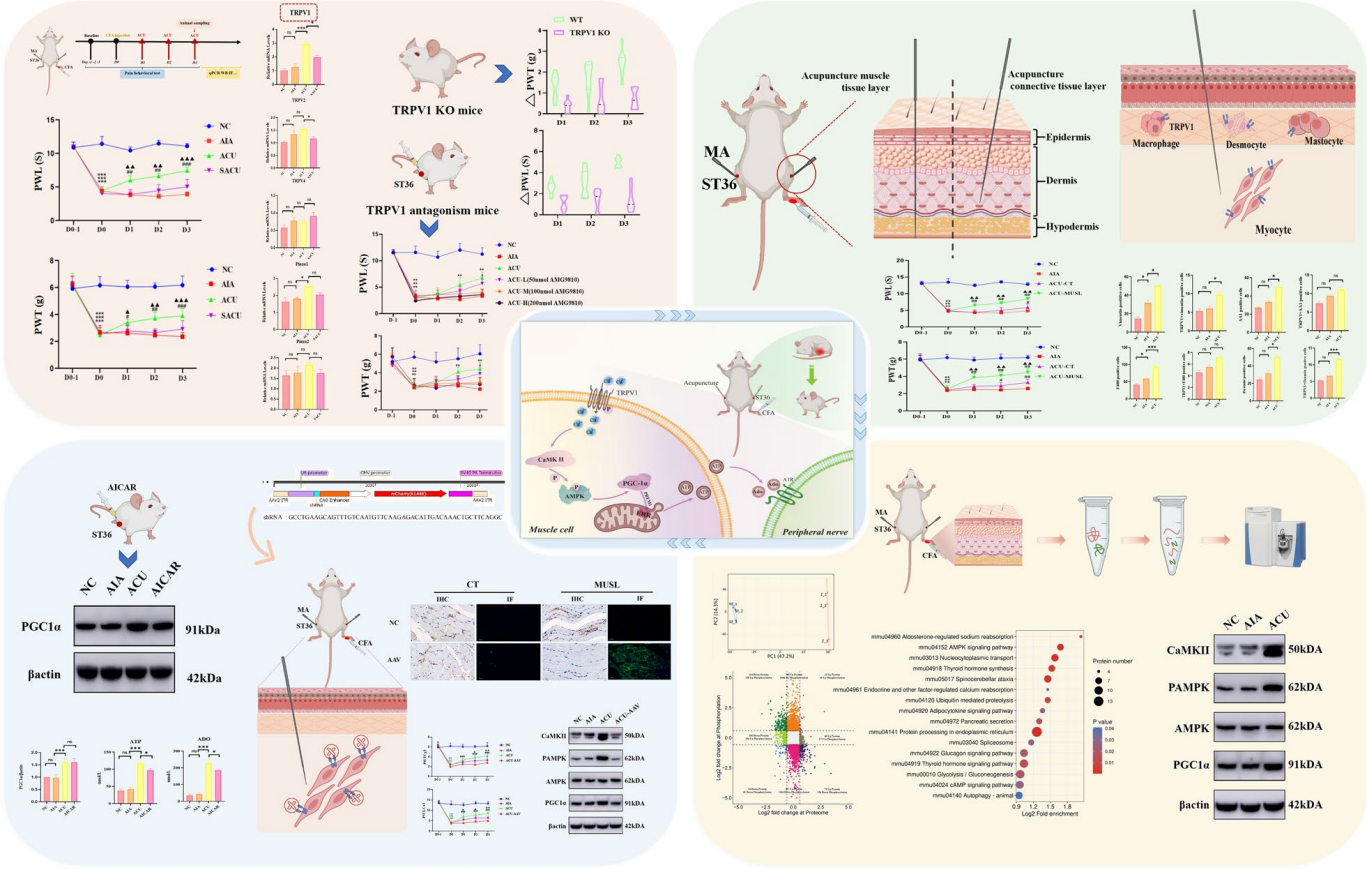

## Introduction

Pain is a complex symptom that covers sensory and emotional dimensions and is an important global public health problem [1]. When acute pain is not well treated, about 20% of patients with acute pain will turn to chronic pain. Inflammatory pain is a very common chronic pain in clinical practice [2]. Some joint, bone diseases and visceral diseases are often accompanied by intolerable inflammatory pain [3]. There are two aspects of the mechanism of inflammatory pain. One is that the release of inflammatory mediators within the tissue continuously stimulates the nociceptors to cause pain in the human body. Second, the increased release of neurotransmitters and up-regulation of receptors at the central end of afferent fibers cause changes in the excitability of sensory neurons in the spinal cord. At present, the drugs for the treatment of inflammatory pain mainly include non-steroidal anti-inflammatory drugs and opioids, but they are often accompanied by great side effects [4]. Traditional Chinese medicine has a unique theoretical system for the understanding of the etiology and pathogenesis of pain. According to the core pathogenesis of'Huangdi Neijing','pain due to obstruction'and'pain due to lack of glory', traditional Chinese medicine believes that the generation of pain is essentially the result of dystrophy or block of viscera and meridians caused by abnormal operation of qi and blood. The treatment of pain in traditional Chinese medicine takes'harmonizing qi and blood, dredging meridians'as the core, and advocates syndrome differentiation. The therapeutic principle emphasizes the regulation of yin and yang, strengthening the body and dispelling evil, tonifying qi and blood in deficiency, dredging collaterals and dispelling evil in reality, and taking into account regional and physical differences. The treatment mainly includes the internal treatment based on the combination of dredging collaterals, tonifying deficiency and warming and clearing, and the external treatment based on acupuncture, massage and external application of traditional Chinese medicine. Through multiple ways to restore the smooth flow of qi and blood, to achieve the treatment of both symptoms and root causes.

Acupuncture is an important component of traditional Chinese medicine [5]. The essence of acupuncture is to insert a needle into the patient’s body at a certain angle, and use acupuncture methods such as twisting and lifting to stimulate specific parts of the human body to achieve the purpose of treating diseases [6]. At the local point, how the body perceives acupuncture stimulation, converts acupuncture information into biological signals, and realizes the distal effect is the key link of acupuncture onset. It has been reported that the local area of Zusanli (ST36) acupoint is rich in PROKR2 neurons [7], which can be activated by acupuncture stimulation to transmit acupuncture information to the central nervous system. And acupuncture stimulation can activate the acupoint local mast cell degranulation [8], increase the release of local histamine, SP, 5-HT, ATP, IL-2, cell chemokines and other factors [9]. In addition, the direction of collagen fibers is radioactively distributed around the acupuncture point, and the morphology of fibroblasts attached to it also changes, affecting the reconstruction of cytoskeleton [10, 11]. At the same time, acupuncture increases the intracellular calcium concentration, initiates the contraction mechanism, and enhances the muscle contraction ability [12]. Although a large number of studies have been conducted on the changes in the microenvironment of acupuncture points, it is still unclear how different types of cells perceive acupuncture stimulation and whether other cells in the acupuncture points are involved in the mechanism of acupuncture signal initiation. In addition, ATP and adenosine have also been shown to play an important role in the initiation of local acupuncture signals at acupoints. However, it is still unclear which cells produce ATP and adenosine [13].

Transient Receptor Potential Vanilloid 1 (TRPV1) is a six-transmembrane protein, mainly expressed in dorsal root ganglion (DRG), spinal cord (SC), brain, skin and other tissues, and also expressed in non-neural cells, such as macrophages, mast cells, fibroblasts, smooth muscle and other cell membranes [14].TRPV1 can be activated by physical and chemical stimuli such as high temperature (> 43 ℃), low pH (< 5.9), as well as endogenous lipid molecules and exogenous ligands such as capsaicin [15]. When activated, the channel opens, leading to the influx of cations such as calcium ions, which activates CaMKII, and CaMKII activation can regulate the function of TRPV1 [16]. It can change the sensitivity and open probability of its channel by phosphorylating TRPV1 channel protein. This phosphorylation modification can change the reactivity of TRPV1 to subsequent stimuli, enhancing or weakening its ability to perceive chemical or physical stimuli [17]. In chronic inflammation, persistent activation of TRPV1 promotes neurogenic inflammation and forms a pain-inflammation vicious cycle. After nerve injury in neuropathic pain, TRPV1 is up-regulated in dorsal root ganglion and spinal dorsal horn, mediating central sensitization and chronic pain maintenance. The excessive activation of TRPV1 in gastrointestinal tract, bladder and other internal organs is closely related to visceral pain. Pharmacological and genetic studies have validated TRPV1 as a therapeutic target in several preclinical models of chronic pain, including cancer, neuropathic, postoperative and musculoskeletal pain [18]. Studies have found that adenosine systemic administration and concentrated administration can produce analgesic effects in various rodent pain models [19]. Activation of TRPV1 in the spinal cord and periphery promotes the release of nucleoside adenosine [20]. Possibly by increasing Ca^2+^ entering the cell through the TRPV1 channel [21]. At the same time, the influx of cations such as calcium ions can cause changes in intracellular energy metabolism. AMPK is an important energy sensor in cells. Studies have found that AMPK is activated when the intracellular ATP/AMP ratio decreases [22]. In addition, PGC1α is a key regulator of mitochondrial biogenesis and metabolism. AMPK activation can phosphorylate PGC1α, enhance its activity, promote mitochondrial biogenesis and fatty acid oxidation and other metabolic processes, and increase energy production [23].

In this study, we found that acupuncture at ST36 acupoint deep needling (muscle layer) is more effective than shallow needling. Through proteomics and genetic identification, it was found that TRPV1 plays an important role in the initiation of acupuncture effect, and it was clarified that local ATP energy metabolism depends on the activation of TRPV1 channel by acupuncture in muscle cells. It provides a new scientific basis for the initiation mechanism of acupuncture point effect.

## Materials and methods

### Animals

This study employed specific pathogen free (SPF)-grade C57BL/6 J adult male wild type (WT) mice (aged 7–8 weeks, weighing 20–22 g) and TRPV1 knockout (KO) mice. WT mice were purchased from Beijing Vital River Laboratory Animal Technology Co., Ltd., while KO mice originated from the laboratory of Shanghai Jiao Tong University [24]. All the mice were housed at the Experimental Animal Center of Tianjin University of Traditional Chinese Medicine for breeding and experimental use. The mice were housed in standard cages (4 per cage) with dimensions of 32.0 cm in length, 21.0 cm in width, and 16.0 cm in height lined with sterilized corncob bedding. The feeding environment was maintained under a 12 h day‒night light cycle (8:00 AM to 8:00 PM), with the humidity controlled at 55–65%. The mice had free access to water and standard feed. All the experimental procedures strictly adhered to the “Guide for the Care and Use of Laboratory Animals” and were approved by the Experimental Animal Ethics Committee of Tianjin University of Traditional Chinese Medicine (Approval No. TCM-LAEC2021279).

### Gene identification

Genomic DNA was extracted via a small rapid extraction kit (BOSTER, MK041-3, Wuhan, China). Polymerase chain reaction (PCR) was performed via a 25-µl reaction system consisting of 12.5 µl of 2xTaq Master Mix (Novoprotein, E005-02B, Wuhan, China), 0.5 µl of F-Prime, 0.5 µl of R-Prime (Sangon, Shanghai, China), 1 µl of DNA, and 10.5 µl of ddH2O (BOSTER, BL510A, Wuhan, China). For the TRPV1 gene (F-Prime: TGCCTCATATTTGCCTTCAG, R-Prime: TGCCTCATATTTGCCTTCAG), the target fragment sizes were 289 bp for wild type (WT), 176 bp for knockout (KO), and 176 bp and 289 bp for heterozygotes. PCR was conducted under the following conditions: initial denaturation at 94 °C for 2 min; 9 cycles of 94 °C for 20 s, 65 °C for 15 s, and 68 °C for 10 s; 27 cycles of 94 °C for 15 s, 60 °C for 15 s, and 72 °C for 10 s; and a final extension at 72 °C for 2 min and holding at 4 °C. Genotype comparison analysis was performed via a gel imaging analysis system (Bio-Rad, California, USA).

### Establishment of the inflammatory pain model

All the mice, except those in the control group, underwent model establishment. The control group of mice received an intradermal injection of 0.9% saline (0.05 mL), whereas the model and acupuncture groups received a subcutaneous injection of complete Freund's adjuvant (CFA) (0.05 mL, 1 mg/mL, Sigma, St. Louis, MO, USA) into the plantar surface of the right hind paw to induce inflammation. For the KO experiments, CFA solution (0.05 mL, Sigma, St. Louis, MO, USA) was also injected into the plantar surface of the right hind paw to induce inflammation. Symptoms such as redness, swelling, decreased movement, and limping of the right foot indicated successful model establishment.

### Acupuncture procedure

In accordance with previous research, acupuncture was performed on the ST36 acupoints on both sides in the acupuncture group via disposable sterile acupuncture needles (0.16 mm diameter, 7 mm length) to a depth of 3 mm. The needles were stimulated via a twisting technique (180° twist, 180 times/minute) for 2 min each, with acupuncture administered once every 5 min for a total of 4 times. The total treatment duration was 28 min. The sham acupuncture group did not receive the twisting technique, and acupuncture was performed 2 mm beside the ST36 acupoint (a nonacupoint). Acupuncture treatment was conducted once daily for 3 consecutive days.

### Acupoint injection of drugs

AMG9810 (GLPBIO, GC11926, Shanghai, China), a TRPV1 antagonist, was injected into the ST36 acupoint at concentrations of 50 μmol, 100 μmol, and 200 μmol. The solvent group received an injection of 10% DMSO + 90% (20% SBE saline) solution. A microinjector was used to puncture the ST36 acupoint, and the drug was slowly injected in multiple directions. Collagenase Type I (Sigma, V900891, St. Louis, MO, USA) was used to degrade the collagen fibers, which were then injected at a concentration of 2.5 mg/mL (20 μL) into the ST36 acupoint. As a control, the solvent group for collagenase type I received an injection of the same volume of TESCA buffer. Succinylcholine chloride dihydrate (CAS6101-15–1, Shanghai, China), a muscle relaxant, was injected at a concentration of 2.5 mg/mL (20 μL) into the local area of the ST36 acupoint. As a control, the solvent group for the muscle relaxant received an injection of the same volume of 0.9% sodium chloride solution (saline). AICAR solution (Sigma‒Aldrich), an AMPK activator, was injected into the ST36 acupoint at a concentration of 5 mg/kg. As a control, the solvent group received an injection of the same volume of 0.9% sodium chloride solution (saline).

### Behavioral tests

A BME-410C thermal radiation pain tester (Ugo Basile, Italy) was used to measure the plantar thermal pain threshold. Testing was conducted in a quiet environment with natural lighting (temperature: 25 ± 2 °C, humidity: 55 ± 5%). The mice were acclimated on transparent grids for 30 min before testing. The thermal pain threshold was defined as the time interval from the start of irradiation to the mouse's pain avoidance response (e.g., paw withdrawal). The average of three measurements was recorded (unit: s). Additionally, mechanical pain thresholds were measured via an electromechanical analgesiometer, and the mechanical force (unit: g) required for the mouse to exhibit a paw withdrawal or escape response was recorded.

### RT-qPCR

RNA samples were extracted via the E.Z.N.A.® HP Total RNA Kit (Omega BioTek, USA). Total RNA was reverse transcribed via the PrimeScript RT Master Mix (Takara Bio, Inc., Otsu, Japan) according to the manufacturer's instructions. RT‒qPCR was then performed on an ABI 7500 machine (Applied Biosystems, Foster City, CA, USA) with SYBR Premix Ex TaqTM (Takara Bio, Inc., Otsu, Japan) according to the manufacturer's instructions. The PCR primers used were designed by Shanghai Sangon Biological Engineering Technology & Services Co., Ltd. and are listed in Table 1. Relative gene expression levels were calculated via the 2^−△△Ct^ method.

**Table 1:** List of primers used

Gene name	Forward primer	Reverse primer
TRPV1	GGCTGTCTTCATCATCCTGCTGCT	GTTCTTGCTCTCCTGTGCGATCTTGT
TRPV2	CTGTCAACAGCGTTGCCACTGA	TTGGTGCCAACTTTCAGCAGCC
TRPV4	CCTTGTTCGACTACGGCACTT	GGATGGGCCGATTGAAGACTT
Piezo1	CTCTACTGGCTGTTGCTGCC	AGGCTACCGTTTTGTCCCAG
Piezo2	GCCAAAGTCAATGGTCGC	GGGTGGGCCAACCTATTTATT
Gapdh	CAAGTTCAACGGCACAGTCAA	CGCCAGTAGACTCCACGACA

### Western blotting (WB)

Proteins were extracted from the muscle tissue of the ST36 acupoint region in mice via RIPA lysis buffer (Beyotime, China), and their concentrations were determined via a BCA protein assay kit. The Membrane Protein Extraction Kit (89,842, USA) was purchased from Thermo Scientific. Samples containing equal amounts of protein were separated via SDS‒PAGE (Bio‒Rad, USA) and then transferred to PVDF membranes (Immobilon‒P, IPVH00010, USA). The membranes were incubated overnight with specific primary antibodies against the following proteins: TRPV1 (ab6166, Abcam, 1:2000, USA), pTRPV1 (12,789, SAB, 1:1000, USA), CaMKII (ab134041, Abcam, 1:2000, USA), AMPK (ab207442, Abcam, 1:1000, USA), pAMPK (ab133448, Abcam, 1:2000, USA), PGC1α (ab191838, Abcam, 1:2000, USA), and β-actin (52,901, SAB, 1:10,000, USA). IgG (H + L) secondary antibody (C31460100, 1:10,000) was purchased from Invitrogen (USA) and incubated at room temperature for 1 h. Finally, an enhanced chemiluminescence (ECL) reagent (AK10129, Elabscience, Wuhan, China) was used as the substrate for development.

### ELISA

ELISA kits (catalog no. YJ925893, YJ963520, Mlbio, Shanghai, China) were used to measure the expression levels of ATP and ADO in the supernatant of lysed muscle tissue from the acupoint region in mice, following the manufacturer’s instructions.

### Immunohistochemistry (IHC)

After isoflurane inhalation anesthesia, the mice were perfused with physiological saline and paraformaldehyde until they were rigid. A 2 × 2 mm tissue block with a depth of 2–3 mm was taken from the acupoint region and rinsed with PBS. The tissue was fixed in 4% paraformaldehyde for 24 h, embedded in paraffin, and sectioned. The sections were dewaxed, incubated with an endogenous peroxidase blocker for 10 min, subjected to EDTA antigen retrieval, and blocked with 5% BSA for 30 min. The primary antibody against TRPV1 (ab305178, Abcam, 1:200, USA) was applied, and the samples were incubated overnight at 4 °C, followed by washing with PBS. The secondary antibody used was polymer anti-rabbit IgG-HRP (ab150079, Abcam, 1:100, USA), and the samples were incubated at room temperature for 30 min and then washed with PBS. DAB staining was performed, followed by incubation with Mayer's hematoxylin at 37 °C for 1 min. After washing with PBS, the sections were mounted with neutral gum. Observations were made under a microscope at 20 × and 40 × magnification, with positive cells appearing brown yellow.

### Immunofluorescence (IF)

For primary antibody incubation, an antibody mixture (AR1016, BOSTER, Wuhan, China) was used to dilute the primary antibodies. On the basis of double standard matching, only TRPV1 (ab305178, Abcam, USA, 1:100) was used for single labeling in the determined layered experiment. To identify the specific cells in which TRPV1 participates in the acupuncture analgesia process, TRPV1 (ab305178, Abcam, 1:100, USA) was mixed with primary antibodies against vimentin (ab92547, Abcam, 1:200, USA), F480 (ab6640, Abcam, 1:200, USA), AA1 (ab2378, Abcam, 1:200,000, USA), and Desmin (Ab15200, Abcam, 1:200, USA) pairwise and incubated overnight at 4 °C. After retrieval, the tissues were incubated at 37 °C for 30 min and washed three times with PBS for 5 min each. For fluorescent secondary antibody incubation, a mixture of Alexa Fluor 488-labeled goat anti-rabbit IgG (Ab150077, Abcam, 1:500, USA) and Alexa Fluor 647-labeled goat anti-mouse IgG (Ab150115, Abcam, 1:500, USA) was added to the tissues and incubated at 37 °C for 45 min. The tissues were then washed three times with PBS (pH 7.2–7.6) for 5 min each. DAPI, a commonly used blue fluorescent DNA dye that can penetrate cell membranes, was applied by adding DAPI staining solution (AR1176, BOSTER, Wuhan, China) to the slides, which were subsequently incubated at room temperature for 3 min, followed by washing with PBS. Anti-fade mounting medium (AR1109, BOSTER, Wuhan, China) was used. The results were observed and photographed via a fluorescence microscope. Under the microscope, the TRPV1 protein appeared as green fluorescence, whereas mast cells, macrophages, fibroblasts, and muscle cells appeared as red fluorescence. After merging via ImageJ 1.8.0 software, the yellow fluorescent areas in the images represented costained cells. Three fields of view were randomly captured for each sample. ImageJ 1.8.0 software was used to count the cells in both channels to obtain the regions of interest (ROIs) for the cells in the two channels. The overlapping ROIs of the two channels were then calculated, and the cells were counted.

### Protein and phosphoprotein profiling

Firstly, the samples from the ST36 acupoint area were taken out from − 80 °C. An appropriate amount of samples was weighed and placed into a mortar pre-chilled with liquid nitrogen, and then ground into powder with the addition of liquid nitrogen. Each sample was added with 4 times the volume of lysis buffer containing 1% Triton X-100, 1% protease inhibitor and 1% phosphatase inhibitor, and then sonicated for lysis. After centrifugation at 12000*g* for 10 min at 4 °C, the supernatant was taken out and the protein concentration was measured using a BCA kit.

Next, equal amounts of proteins from each sample were taken for enzymatic digestion. The volume was adjusted to be consistent with the lysis buffer. Then, trichloroacetic acid (TCA) was added to a final concentration of 20%, and the mixture was vortexed evenly and precipitated at 4 °C for 2 h. After centrifugation at 4500*g* for 5 min, the supernatant was discarded, and the precipitate was washed with pre-chilled acetone for 2–3 times. After drying the precipitate, 200 mM TEAB was added to a final concentration, and the precipitate was dispersed by sonication. Trypsin was added at a ratio of 1:50, and the enzymatic digestion was carried out overnight. Subsequently, dithiothreitol (DTT) was added to a final concentration of 5 mM, and the mixture was reduced at 56 °C for 30 min. Then, iodoacetamide (IAA) was added to a final concentration of 11 mM, and the mixture was incubated at room temperature in the dark for 15 min.

After that, the peptides were dissolved in the enrichment buffer solution (50% acetonitrile/0.5% acetic acid). The supernatant was transferred to the pre-washed IMAC materials and incubated on a rotary shaker with gentle shaking. Subsequently, the materials were washed 3 times successively with buffer solutions of 50% acetonitrile/0.5% acetic acid and 30% acetonitrile/0.1% trifluoroacetic acid. Finally, the phosphopeptides were eluted with 10% ammonia water, and the eluate was collected and freeze-dried in a vacuum. After drying, desalting was carried out according to the instructions of C18 ZipTips, and after vacuum freeze-drying again, it was provided for liquid chromatography-mass spectrometry (LC–MS) analysis. HPLC fractionation was not performed in this project. During the LC–MS analysis, the peptides were dissolved in mobile phase A containing 0.1% formic acid and 2% acetonitrile, and then separated by the NanoElute ultra-high-performance liquid system. Mobile phase B was an acetonitrile–water solution containing 0.1% formic acid. The liquid chromatographic gradient was set as follows: 0–16 min, 2–22% B; 16–22 min, 22–35% B; 22–26 min, 35–90% B; 26–30 min, 90% B, and the flow rate was maintained at 450 nl/min. After separation, the peptides were injected into the Capillary ion source for ionization and then entered the timsTOF Pro mass spectrometer for data acquisition. The ion source voltage was set at 1.7 kV. The data acquisition mode used the data-independent parallel accumulation serial fragmentation (dia-PASEF) mode. The scanning range of the primary mass spectrum was set from 100 to 1700 m/z. After one primary mass spectrum was collected, 22 PASEF mode acquisitions were carried out. The scanning range of the secondary mass spectrum was from 395 to 1395, with a window of every 20 m/z.

In terms of functional analysis, with the identified proteins as the background, the Kyoto Encyclopedia of Genes and Genomes (KEGG) pathway enrichment analysis was conducted by using the corresponding database. The Fisher's exact test was used to determine the significance of pathway enrichment of the differentially expressed modified proteins. P < 0.05 was considered significant.

### Data analysis

The data are expressed as the means ± standard errors of the means (SEMs) and were statistically analyzed via SPSS 21.0 software. For normally distributed data, one-way ANOVA was used; for nonnormally distributed data, nonparametric tests were applied. Under the premise of a normal distribution and homogeneity of variance, the least significant difference (LSD) test or Dunnett’s T3 test was used for multiple comparisons. Repeated measures analysis was employed for multiple datasets. P < 0.05 was considered statistically significant, P < 0.01 was considered highly statistically significant, and p < 0.001 was considered extremely statistically significant.

## Result

### Acupuncture alleviates inflammatory pain in mice

**Fig. 1:**
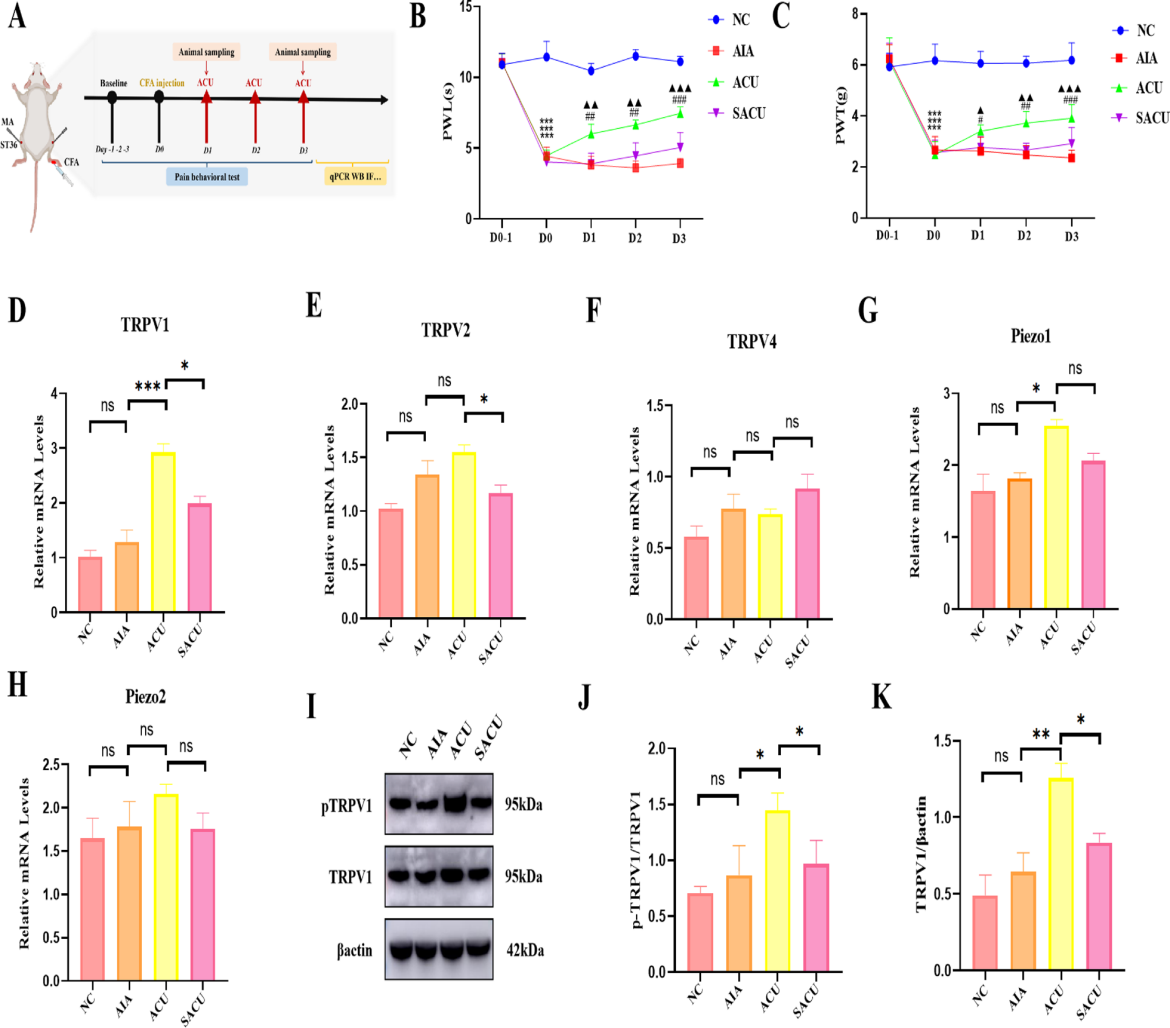
Acupuncture alleviates inflammatory pain and increased TRPV1 expression at ST36 points in mice. **A** Flowchart of the modeling process and measurement indicators in AIA mice. **B** Effect of acupuncture on the paw withdrawal latency (PWL) of mice induced by complete Freund's adjuvant (CFA) (n = 8 per group). **C** Effect of acupuncture on the paw withdrawal threshold (PWT) of mice induced by CFA (n = 8 per group). **D**–**H** Gene expressions of TRPV1, TRPV2, TRPV4, Piezo1 and Piezo2 at the ST36 acupoint (n = 3 per group). **I** Protein expressions of TRPV1 and pTRPV1 at the ST36 acupoint (n = 3 per group). **J** Quantitative diagram of pTRPV1. **K** Quantitative diagram of TRPV1.Data are presented as the mean ± SD. *p < 0.05, **p < 0.01, ***p < 0.001

We assessed the degree of hyperalgesia induced by Complete Freund's Adjuvant (CFA) injection and the analgesic effect of acupuncture by measuring paw withdrawal latency (PWL) to thermal stimuli and paw withdrawal threshold (PWT) to mechanical stimuli. Compared with the control group, the pain thresholds of mice in the AIA (arthritis-induced model) group were reduced on days 1, 2 and 3, indicating successful model establishment. Compared with the AIA group, the PWL and PWT of mice in the ACU (acupuncture) group were significantly increased on days 1, 2 and 3, while no significant increase was observed in the sham acupuncture group (p < 0.01, Fig. 1A–C), suggesting that acupuncture exerts an analgesic effect.

### Acupuncture can increases TRPV1 expression at ST36

We performed qPCR analysis on ion channels related to acupuncture at the ST36 acupoint area, including TRPV1, TRPV2, TRPV4, Piezo1 and Piezo2. The results showed that the mRNA expression levels of TRPV1 and Piezo1 in the ACU group were higher than those in the NC (normal control) and AIA groups, with TRPV1 showing the most significant increase (p < 0.001, Fig. 1D–H). Since TRPV1 is mainly expressed on the cell membrane, we extracted membrane proteins from the acupoint tissues and detected TRPV1 membrane protein expression and activity through Western blotting (WB) experiments. It was found that compared with the AIA group, the phosphorylated TRPV1 (PTRPV1) and TRPV1 expression levels were significantly upregulated in the ACU group, indicating that acupuncture can increase TRPV1 expression and activity at the acupoint area (p < 0.01, Fig. 1I–K).

### TRPV1 mediates the analgesic effect of acupuncture

**Fig. 2:**
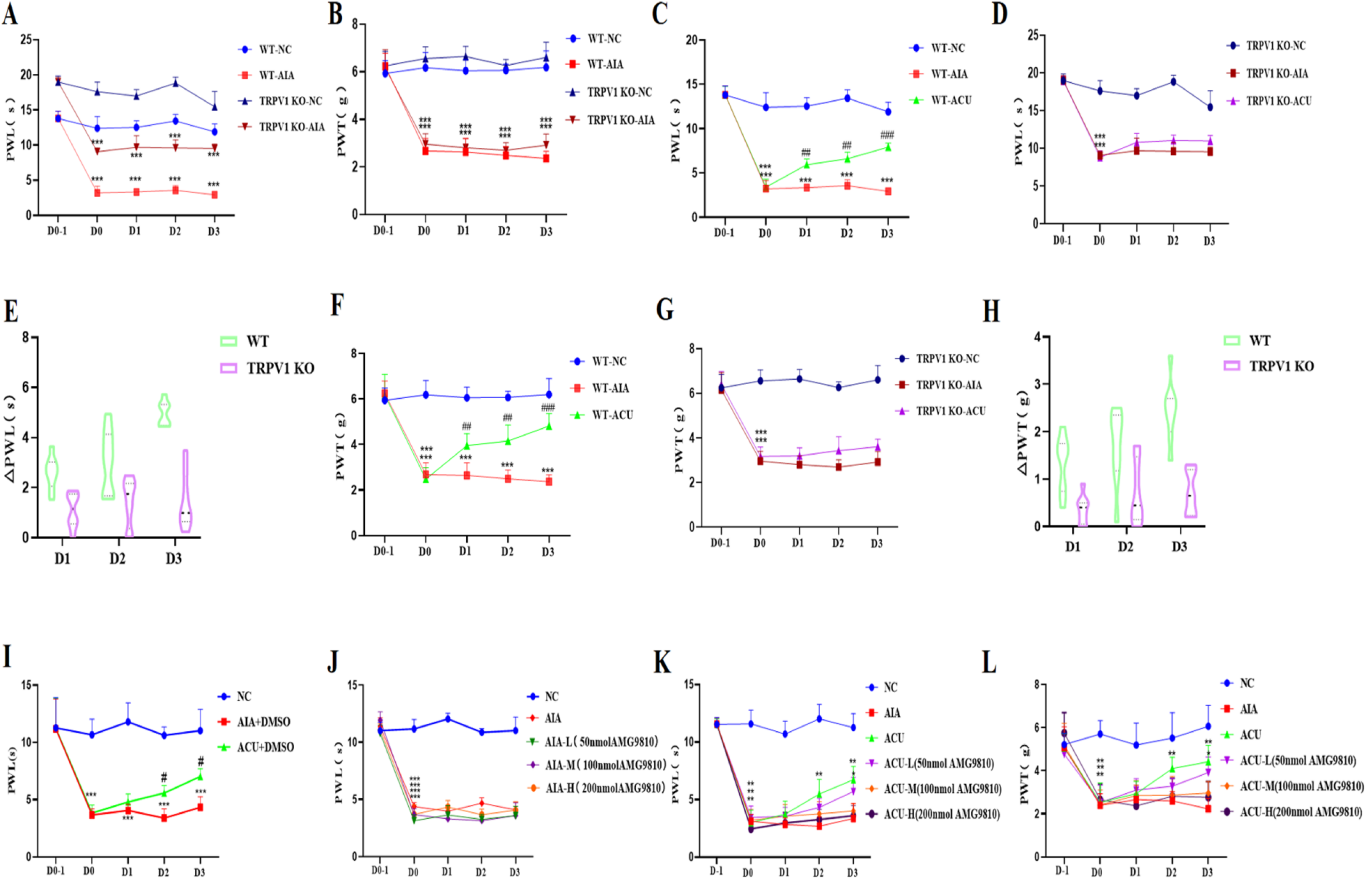
TRPV1 in the ST36 acupoint area mediates the analgesic effect of acupuncture. **A** Paw withdrawal latency (PWL) of TRPV1 knockout (KO) and wild-type (WT) mice induced by CFA (n = 8 per group). **B** Paw withdrawal threshold (PWT) of TRPV1 KO and WT mice induced by CFA (n = 8 per group). **C**, **D** Effect of acupuncture on the PWL of TRPV1 KO and WT mice induced by CFA (n = 8 per group). **E** Quantitative analysis and comparison of the effect of acupuncture on the PWL of TRPV1 KO and WT mice induced by CFA (n = 8 per group). **F**–**G** Effect of acupuncture on the PWT of TRPV1 KO and WT mice induced by CFA (n = 8 per group). **H** Comparison of the effect of acupuncture on the PWT of TRPV1 KO and WT mice induced by CFA (n = 8 per group). **I** Effect of the solvent DMSO of TRPV1 antagonist AMG9810 on the analgesic effect of acupuncture. **J** Effect of AMG9810 on the modeling of CFA mice (n = 6 per group). **K**, **L** Inhibitory effect of different doses of AMG9810 on the analgesic effect of acupuncture (PWL & PWT) (n = 6 per group). Data are presented as the mean ± SD. *p < 0.5. **p < 0.05. ***p < 0.01

Our research data revealed that TRPV1 expression at the ST36 acupoint was significantly upregulated after acupuncture intervention. Based on this, we further explored the potential impact of TRPV1 deficiency on the analgesic efficacy of acupuncture. Notably, under baseline conditions, there was a significant difference in PWL between TRPV1 knockout (KO) mice and wild type (WT) mice, with TRPV1 KO mice exhibiting a higher baseline threshold. This difference is attributed to TRPV1 being a highly temperature-sensitive ion channel, making WT mice more sensitive to thermal stimuli. However, no statistically significant difference was observed in baseline PWT between the two groups of mice (p > 0.05, Fig. 2 A and B). Regarding the therapeutic effect, we observed that the pain thresholds of WT mice that received acupuncture treatment were significantly elevated on days 1, 2, and 3 (p < 0.05, Fig. 2 C and F), indicating that acupuncture effectively alleviated pain in these mice. In contrast, mice in the TRPV1 KO group that received acupuncture (TRPV1^−/−^ ACU) did not show significant changes in pain thresholds during the same period (p > 0.05, Fig. 2E and G), suggesting that the absence of TRPV1 may impair the analgesic effect of acupuncture. Specifically, compared with the TRPV1 ACU group, the △PWL of TRPV1^−/−^ ACU mice decreased by 57.7%, 52.8%, and 71% on days 1, 2, and 3, respectively (Fig. 2E), and the △PWT decreased by 60.8%, 57.6%, and 71.9%, respectively (Fig. 2H). These data further confirm the crucial role of TRPV1 in the analgesic mechanism of acupuncture. In summary, our results indicate that TRPV1 is not only an important molecular basis for temperature perception but also an indispensable molecular target for the analgesic effect of acupuncture, and its absence or functional inhibition significantly affects the analgesic efficacy of acupuncture.

### TRPV1 at the ST36 acupoint mediates the analgesic effect of acupuncture

To explore the specific role of TRPV1 at the ST36 acupoint in initiating the effect of acupuncture, we adopted a more precise intervention strategy: local injection of the TRPV1 antagonist AMG9810 at the ST36 acupoint. Prior to this, we rigorously verified that the antagonist solvent DMSO had no effect on the analgesic effect of acupuncture (Fig. 2I) and confirmed that different doses of the antagonist did not interfere with the success rate of model establishment (Fig. 2J), laying a solid foundation for subsequent experiments. Subsequently, we administered antagonist injections at the acupoint, setting up low (50 nmol), medium (100 nmol), and high (200 nmol) dose groups. In the comparative analysis between the AIA group and the control group, we observed significant reductions in PWL and PWT on days 1, 2, and 3 (p < 0.001, Fig. 2K and L), indicating successful model establishment and effective induction of pain sensitization. Further, compared with the AIA group, the group that received acupuncture treatment combined with a low dose of antagonist (ACU + low-dose group) showed significant increases in PWL and PWT on days 2 and 3 (P < 0.01), although this increase was still slightly lower than that in the acupuncture-only group (ACU group) (P < 0.001, Fig. 2K and L). This initially suggests that partial blockade of TRPV1 at the acupoint by a low dose of AMG9810 may facilitate the manifestation of the analgesic effect of acupuncture. However, when acupuncture treatment was combined with medium or high doses of the antagonist (i.e., ACU + medium-dose group and ACU + high-dose group), we observed significant reductions in PWL and PWT on days 1, 2, and 3 compared with the acupuncture-only group (p < 0.01, Fig. 2K and L). This result suggests that TRPV1 at the acupoint plays a vital role in the process of acupuncture analgesia, and excessive inhibition of its function significantly impairs the analgesic effect of acupuncture.

### The muscle layer at ST36 primarily mediates the analgesic effect of acupuncture

**Fig. 3:**
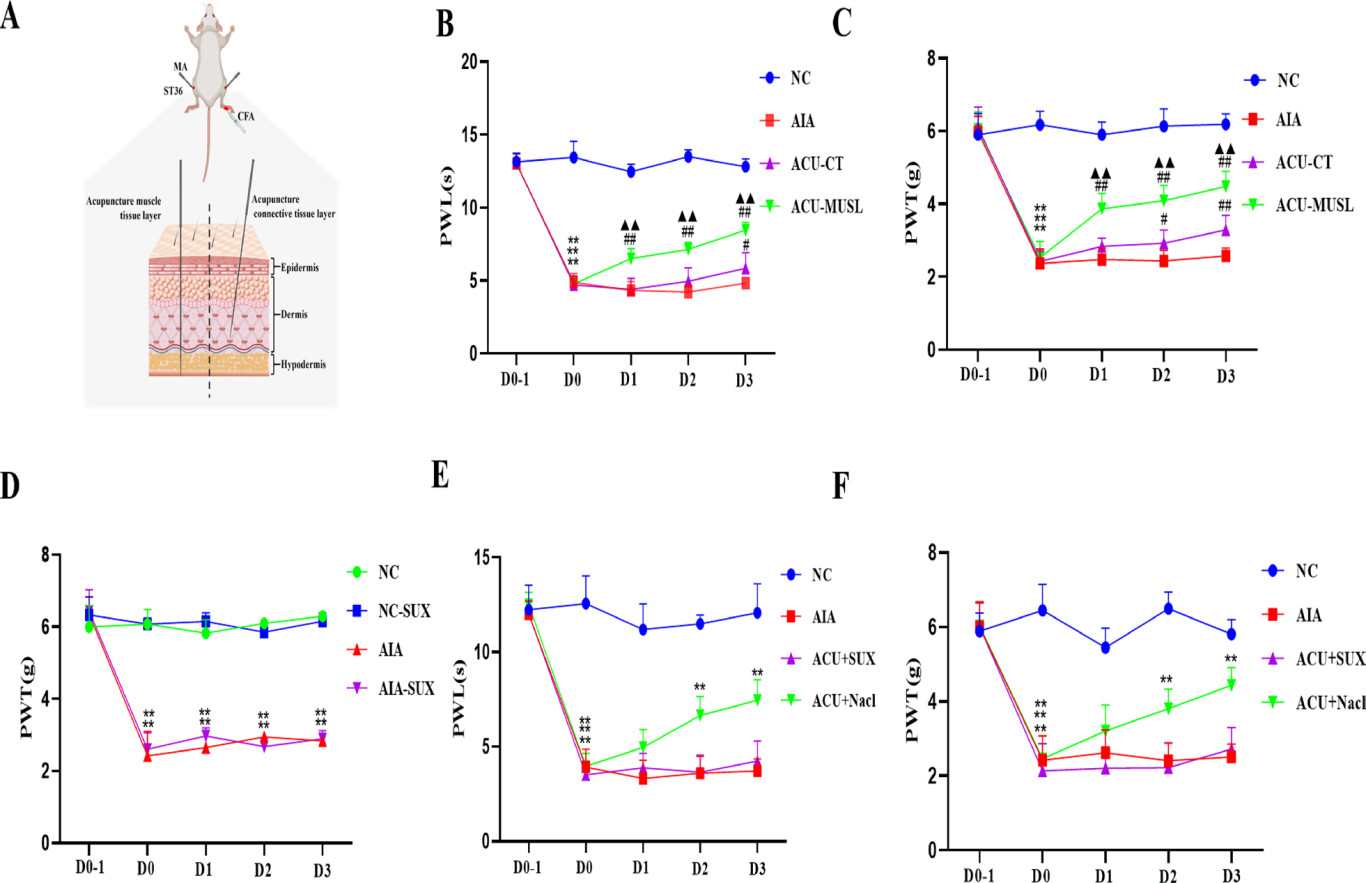
The muscular layer of the acupuncture point area mainly produces the analgesic effect of acupuncture. **A** Schematic diagram of the local superficial and deep layers of the acupuncture point area in mice. **B** Comparison of the analgesic effects of acupuncture on the superficial layer (ACU-CT) and the deep layer (ACU-MUSL) (PWL) (n = 8 per group). **C** Comparison of the analgesic effects of acupuncture on the superficial layer (ACU-CT) and the deep layer (ACU-MUSL) (PWT) (n = 8 per group). **D** Effect of local injection of muscle relaxant (SUX) at the acupoint on the CFA-induced modeling (n = 8 per group). **E** Effect of local injection of muscle relaxant (SUX) at the acupoint on the paw withdrawal latency (PWL) of mice (n = 8 per group). **F** Effect of local injection of muscle relaxant (SUX) at the acupoint on the paw withdrawal threshold (PWT) of mice (n = 8 per group).Data are presented as the mean ± SD. *p < 0.05, **p < 0.01, ***p < 0.001

To further investigate the specific contributions of different layers at the acupoint to the analgesic effect of acupuncture, we designed a controlled experiment to compare the therapeutic effects of acupuncture targeting the superficial fascia layer (ACU + CT group) and acupuncture targeting the deep muscle layer (ACU + MUSL group) (Fig. 3A). In the baseline comparison between the AIA group and the control group, we observed significant reductions in PWL and PWT on days 1, 2, and 3 (p < 0.01), confirming successful model establishment and establishing the baseline state of pain sensitization. Further, we compared the therapeutic differences between the two acupuncture strategies. The results showed that the group receiving acupuncture in the deep muscle layer (ACU + MUSL) exhibited significant increases in PWL and PWT on days 1, 2, and 3 after treatment (P < 0.001), while the group receiving acupuncture in the superficial fascia layer (ACU + CT) only showed mild increases in PWL and PWT on day 3 (p < 0.05, Fig. 3B and C). This finding initially suggests that acupuncture targeting the deep muscle layer may have a more pronounced analgesic effect than targeting the superficial fascia layer, especially in the treatment of inflammatory pain. To verify this conclusion, we further conducted validation experiments using a muscle relaxant as a tool drug. First, we confirmed that the muscle relaxant itself had no significant effect on model establishment (P < 0.01, Fig. 3D), ensuring the validity of the experiment. Subsequently, we compared the acupuncture + muscle relaxant injection group (ACU + SUX) with the acupuncture + muscle relaxant solvent group (ACU + NaCl). The results showed that the PWL and PWT of the ACU + SUX group were significantly lower than those of the ACU + NaCl group on days 1, 2, and 3 after treatment (P < 0.01, Fig. 3E and F). This result supports our hypothesis that the deep muscle layer at the acupoint plays a more central role in the process of acupuncture analgesia.

### TRPV1 in the muscle layer of ST36 primarily mediates acupuncture analgesia

**Fig. 4:**
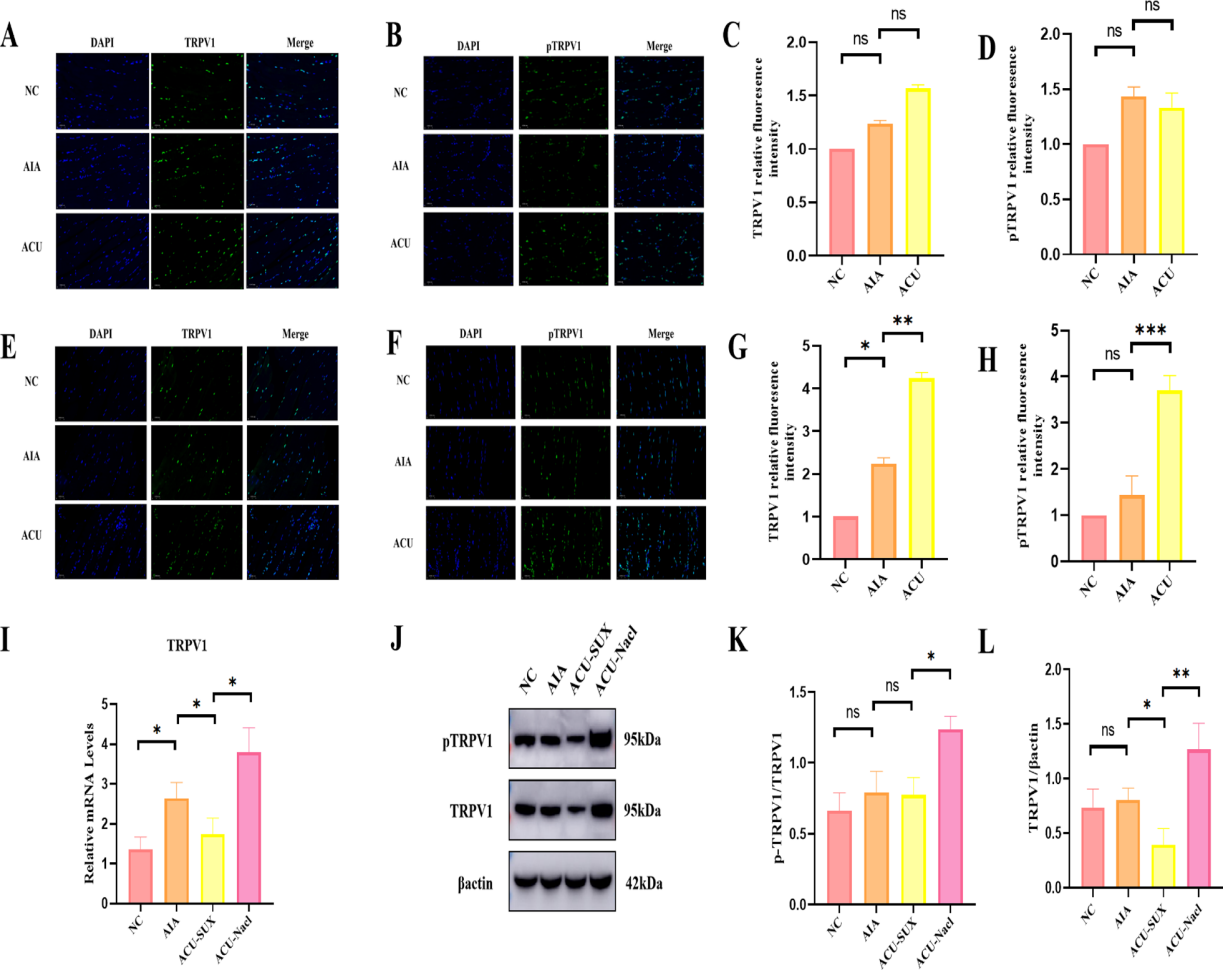
Acupuncture Primarily Increases TRPV1 Expression in the Muscle Layer at ST36. **A**, **B** Immunofluorescence expression of TRPV1 and phosphorylated TRPV1 (pTRPV1) in the local fascial layer at the acupuncture point region in mice after acupuncture (n = 3 per group). **C**, **D** Quantitative analysis of the immunofluorescence expression of TRPV1 and pTRPV1 in the local fascial layer at the acupuncture point region in mice after acupuncture. **E**, **F** Immunofluorescence expression of TRPV1 and pTRPV1 in the local muscle layer at the acupuncture point region in mice after acupuncture (n = 3 per group). **G**, **H** Quantitative analysis of the immunofluorescence expression of TRPV1 and pTRPV1 in the local fascial layer at the acupuncture point region (note: this should likely refer to the muscle layer instead of fascial layer for consistency, but I'll keep it as is for now) in mice after acupuncture. **I** Changes in TRPV1 gene expression after injection of a muscle relaxant (n = 3 per group). **J** Changes in TRPV1 and pTRPV1 protein expression after injection of a muscle relaxant (n = 3 per group). **K**, **L** Quantitative analysis of changes in pTRPV1 and TRPV1 protein expression after injection of a muscle relaxant (n = 3 per group). Data are presented as the mean ± SD. *p < 0.05, **p < 0.01, ***p < 0.001

To delve into the role of TRPV1 in the muscle layer of acupoints in the mechanism of acupuncture analgesia, we conducted a specialized study on TRPV1 using immunofluorescence single-labeling technique. In the experiment, two comparison groups were established: one group received acupuncture at the superficial fascial layer (denoted as ACU + CT group), and the other group received acupuncture at the deep muscle layer (denoted as ACU + MUSL group). By closely observing the changes in the expression and activity of TRPV1 and its phosphorylated form (pTRPV1) before and after acupuncture, we made key findings. The results indicated that in the ACU + CT group, acupuncture did not significantly alter the expression levels of TRPV1 and pTRPV1, with statistical analysis showing no significant difference (p > 0.05, Fig. 4A–D). However, in the ACU + MUSL group, acupuncture at the deep muscle layer significantly upregulated the expression levels of TRPV1 and pTRPV1 (p < 0.001, Fig. 4E–H). This finding suggests that acupuncture at the deep muscle layer is more effective in promoting TRPV1 expression and enhancing its activity compared to the superficial fascial layer.

To further validate this finding and explore the impact of muscle state on TRPV1 expression and acupuncture analgesia, we designed another set of experiments. Using Western Blotting, we compared the protein expression levels of TRPV1 and pTRPV1 in the acupuncture combined with muscle relaxant injection group (ACU + SUX) and the acupuncture combined with muscle relaxant solvent control group (ACU + NaCl). The experimental results showed that the expression levels of TRPV1 and pTRPV1 in the ACU + SUX group were significantly lower than those in the control group(p < 0.01,Fig. 4I–L), indicating that the administration of muscle relaxants not only effectively antagonized the analgesic effect of acupuncture but also inhibited TRPV1 expression and its activity. Our research results demonstrate that TRPV1 in the muscle layer of the ST36 acupoint plays a key role in mediating the analgesic effect of acupuncture. Acupuncture at the deep muscle layer can exert an analgesic effect while significantly enhancing TRPV1 expression and activity, whereas the administration of muscle relaxants can reverse this process, further confirming the central role of TRPV1 in the muscle layer of ST36 in the mechanism of acupuncture analgesia.

### TRPV1 on myocytes in the ST36 acupoint region primarily mediates acupuncture analgesia

**Fig. 5:**
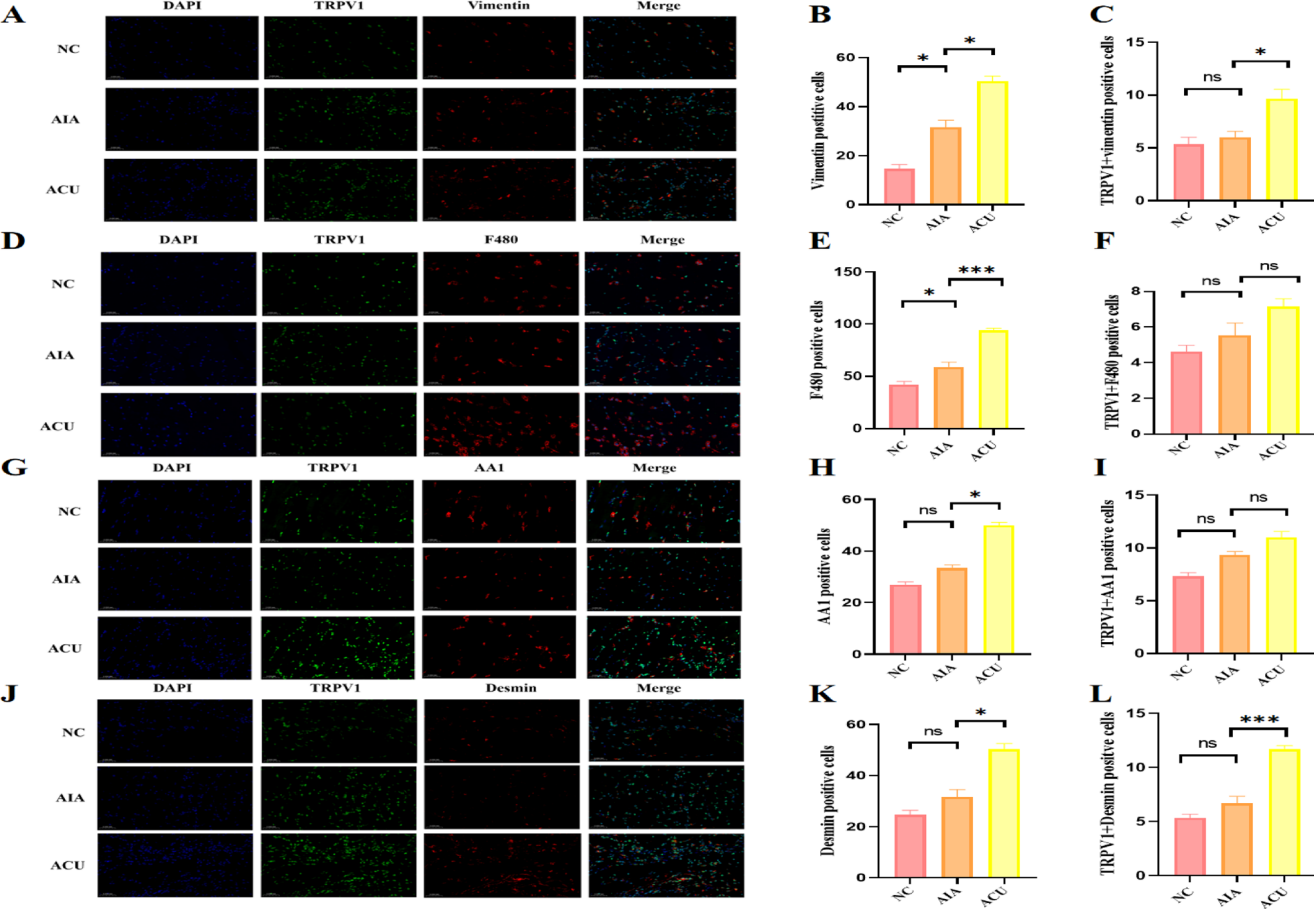
The expression of TRPV1 on ST36 muscle cells increases with acupuncture. **A** Immunofluorescence image of the expression of fibroblasts in the local acupuncture point area of mice after acupuncture (n = 3 per group). **B** Quantitative analysis of the change in the number of fibroblasts. **C** Quantitative analysis of the co-localization of fibroblasts and TRPV1. **D** Immunofluorescence image of the expression of macrophages in the local acupuncture point area of mice after acupuncture (n = 3 per group). **E** Quantitative analysis of the change in the number of macrophages. **F** Quantitative analysis of the co-localization of macrophages and TRPV1. **G** Immunofluorescence image of the expression of mast cells in the local acupuncture point area of mice after acupuncture (n = 3 per group). **H** Quantitative analysis of the change in the number of mast cells. **I** Quantitative analysis of the co-localization of mast cells and TRPV1. **J** Immunofluorescence image of the expression of myoblasts in the local acupuncture point area of mice after acupuncture (n = 3 per group). **K** Quantitative analysis of the change in the number of myoblasts. **L** Quantitative analysis of the co-localization of myoblasts and TRPV1. Data are presented as the mean ± SD. *p < 0.05, **p < 0.01, ***p < 0.001

To precisely define the cell-specific localization of TRPV1 in the acupuncture analgesia effect, we initially employed immunofluorescence double-labeling technique to conduct an in-depth analysis of fibroblasts, macrophages, mast cells, and myocytes, which are known to respond significantly to acupuncture. The experimental results revealed that compared with the AIA group (model control group), the ACU group (acupuncture treatment group) not only significantly promoted fibroblast proliferation (p < 0.05) but also increased the number of TRPV1-positive fibroblasts (p < 0.05, Fig. 1A–C). Although the total number of macrophages in the ACU group was significantly higher than that in the AIA group (p < 0.001), there was no significant change in the number of TRPV1-positive macrophages (p > 0.05, Fig. 5D–F). Similarly, the number of mast cells increased in the ACU group (p < 0.05), yet the number of TRPV1-positive mast cells remained relatively stable (p > 0.05, Fig. 5G–I). Notably, the number of myocytes in the ACU group not only increased (p < 0.05) but also showed a significant rise in the number of TRPV1-positive myocytes (p < 0.001, Fig. 5J–L). It shows that acupuncture induces muscle fiber hypertrophy and promotes the proliferation of satellite cells by activating TRPV1 positive sensory nerve endings and releasing neurotrophic factors, suggesting that myocytes may be the key cell type through which TRPV1 mediates the acupuncture analgesia effect.

### Proteomics and phosphoproteomics unravel the acupuncture-activated local acupoint signaling pathway

**Fig. 6:**
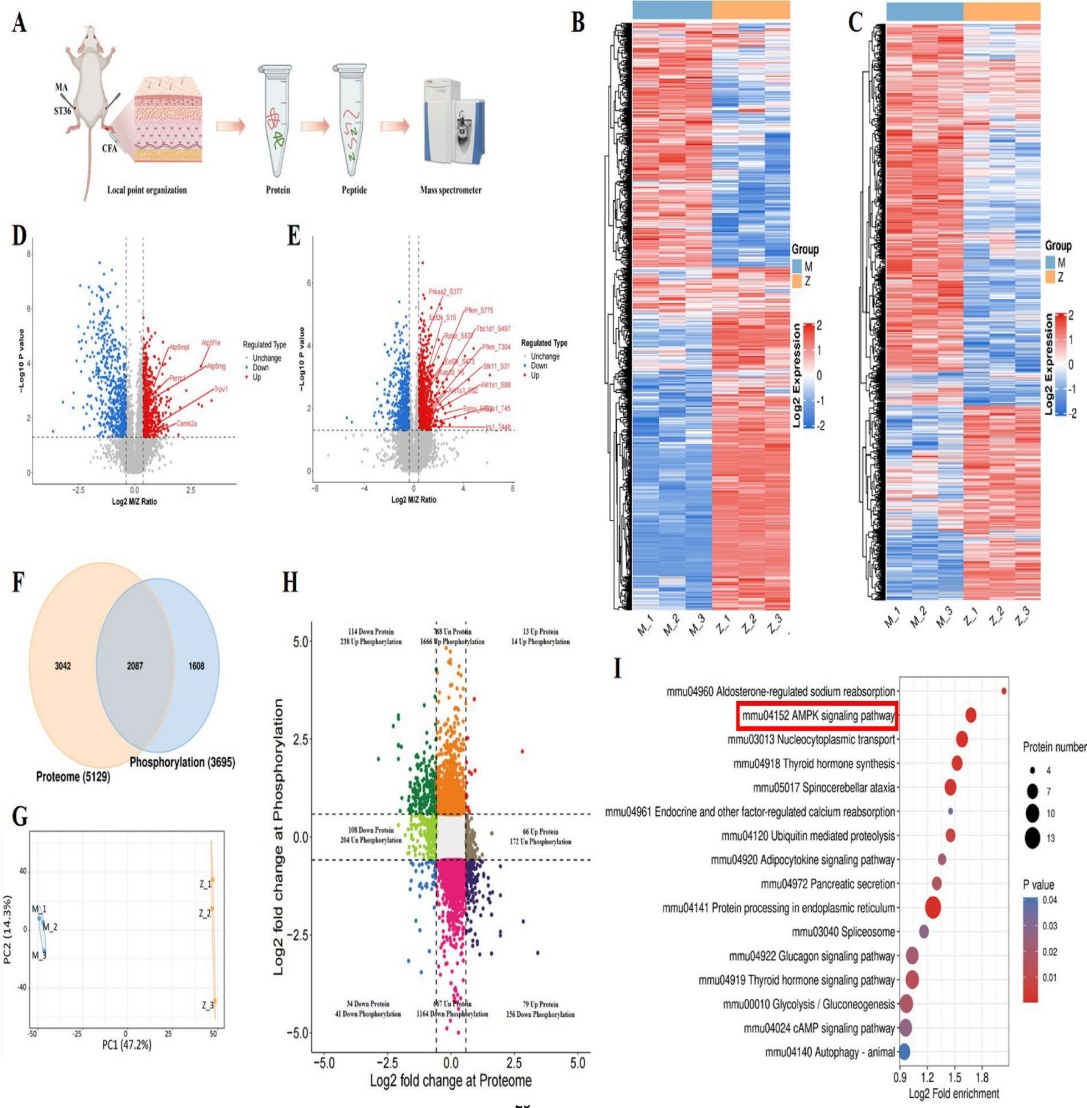
Results based on combined proteomic and phosphoproteomic analysis. **A** Experimental flowchart of proteomics and phosphoproteomics (n = 3 per group). **B** Heatmap of proteomics. **C** Heatmap of phosphoproteomics. **D** Volcano plot of proteomics. **E** Volcano plot of phosphoproteomics. **F** Comparative analysis diagram of combined identification in proteomics and phosphoproteomics. **G** PCA plot of proteomics and phosphoproteomics. **H** Nine-quadrant diagram of combined analysis in proteomics and phosphoproteomics. **I** KEGG pathway diagram of combined analysis in proteomics and phosphoproteomics

In an in-depth exploration of the regulatory mechanisms of acupuncture on muscular transient receptor potential vanilloid subtype 1 (TRPV1), we innovatively integrated advanced techniques from proteomics and phosphoproteomics to conduct a meticulous comparative analysis of local tissues at the ST36 acupoint region between the AIA group (denoted as M) and the ACU group (denoted as Z) (as shown in Fig. 6A). Employing principal component analysis (PCA), we observed a high degree of clustering among the three repeated experimental data points for each sample, robustly validating the stability and reproducibility of our experimental data (see Fig. 6G). Through heatmap visualization, we clearly identified significant differences in protein expression and phosphorylation site modifications between the AIA and ACU groups, both at the proteomic level (Fig. 6B) and the phosphoproteomic level (Fig. 6C). In proteomic analysis, we successfully identified and quantified 5129 proteins; whereas in phosphoproteomic analysis, we further identified 4102 proteins with phosphorylation modifications. A meticulous cross-comparison analysis revealed that 2227 proteins were reliably identified at both levels (see Fig. 6F and H), providing a solid foundation for subsequent analyses. To gain deeper insights into these differences, we conducted differential expression analysis using volcano plots (Fig. 6D). The results showed that a series of key proteins, including TRPV1, calcium/calmodulin-dependent protein kinase II (CaMKII), ATP synthase-related subunits (such as ATP5mpl, Atp5mg, Atp5f1e), and PGC1α-related protein (Perm1), were significantly upregulated at the proteomic level. These upregulations may have crucial implications for the molecular mechanisms of acupuncture effects. Meanwhile, at the phosphoproteomic level (Fig. 6E), we also identified multiple related proteins and key phosphorylation sites in the AMP-activated protein kinase (AMPK) phosphorylation pathway. Through KEGG pathway analysis, we further pinpointed the AMPK signaling pathway as an important target of acupuncture action (Fig. 6I). These findings not only unveil the complex molecular mechanisms underlying acupuncture's regulation of muscular TRPV1 but also provide new perspectives and clues for understanding the widespread effects of acupuncture therapy in biological systems, with significant biological and clinical implications.

### Protein expression validation

**Fig. 7:**
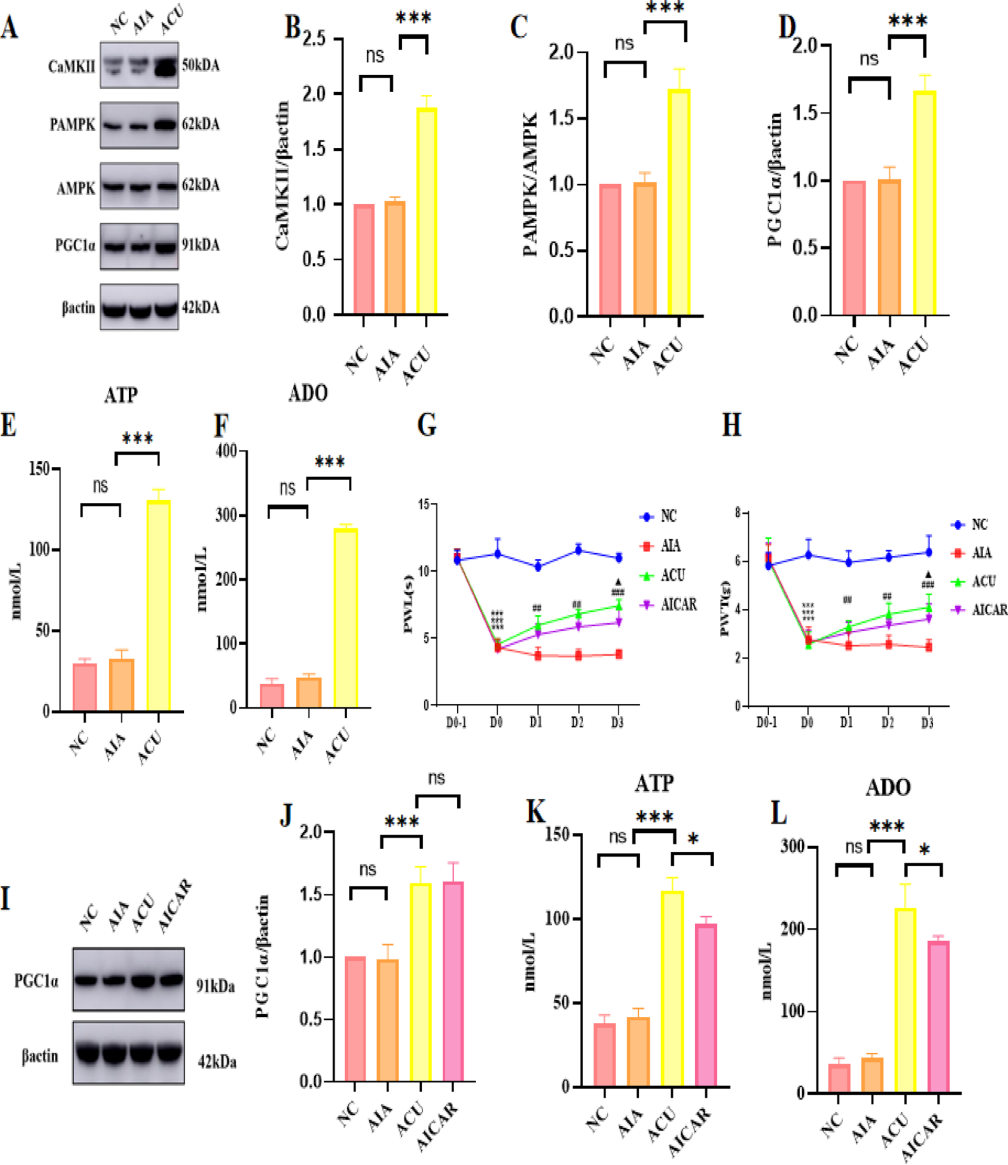
Verification of the screened CaMKII/AMPK/PGC1α signaling pathway. **A** Effect of acupuncture on the protein expression levels of CaMKII, pAMPK, AMPK, and PGC1α at the local acupoint (n = 6 per group). **B**–**D** Quantitative diagrams of the protein expression levels of CaMKII, AMPK phosphorylation, and PGC1α. **E**, **F** Expression levels of ATP and ADO (n = 6 per group). **G**, **H** Effect of local injection of AMPK activator AICAR at the acupoint on pain (PWL and PWT) (n = 6 per group). **I**, **J** Changes in the protein expression of PGC1α and quantitative analysis after acupoint injection of AICAR (n = 6 per group). **K**, **L** Expression levels of ATP and ADO after acupoint injection of AICAR (n = 6 per group). Data are presented as the mean ± SD. *p < 0.05, **p < 0.01, ***p < 0.001

To further validate the expression of key proteins identified in the proteomic analysis, we employed Western Blotting (WB) technique to precisely quantify the protein expression levels of calcium/calmodulin-dependent protein kinase II (CaMKII), phosphorylated AMP-activated protein kinase (pAMPK), AMP-activated protein kinase (AMPK), and PGC1α. The experimental results clearly demonstrated that in the ACU group, the protein expression of CaMKII was significantly increased, the phosphorylation level of AMPK was markedly enhanced, and the protein expression of PGC1α also showed an upward trend (p < 0.001, Fig. 7A–D), These results are highly consistent with our previous joint analysis of proteomics and phosphoproteomics, providing strong support for the reliability of the data. To comprehensively understand the functions and interactions of these proteins, we subsequently utilized Enzyme-Linked Immunosorbent Assay (Elisa) technique to measure the expression levels of ATP and adenosine (ADO) (p < 0.001, Fig. 7E, F). The experimental data indicated that in the ACU group, the expression levels of both ATP and ADO were significantly elevated, which once again aligns with our joint analysis of proteomics and phosphoproteomics.

### Validation of AMPK’s role in acupuncture analgesia pathway

To further confirm the existence and function of this pathway, we adopted the method of locally injecting AICAR, an AMPK activator, into the acupoint and observed the animals'pain responses as well as the expression levels of PGC1α, ATP, and ADO to validate the crucial role of AMPK in this pathway. The experimental results indicated that AICAR injection could mimic the analgesic effect of acupuncture (p < 0.001, Fig. 7G, H), and in the AICAR group, the expression levels of PGC1α, ATP, and ADO were significantly increased (p < 0.001, Fig. 7I–L). These findings robustly demonstrate that the local acupoint signaling pathway CaMKII/AMPK/PGC1α plays a vital role in the analgesic effect of acupuncture.

### Identified CaMKII/AMPK/PGC1α as the downstream pathway of TRPV1 on myocytes

**Fig. 8:**
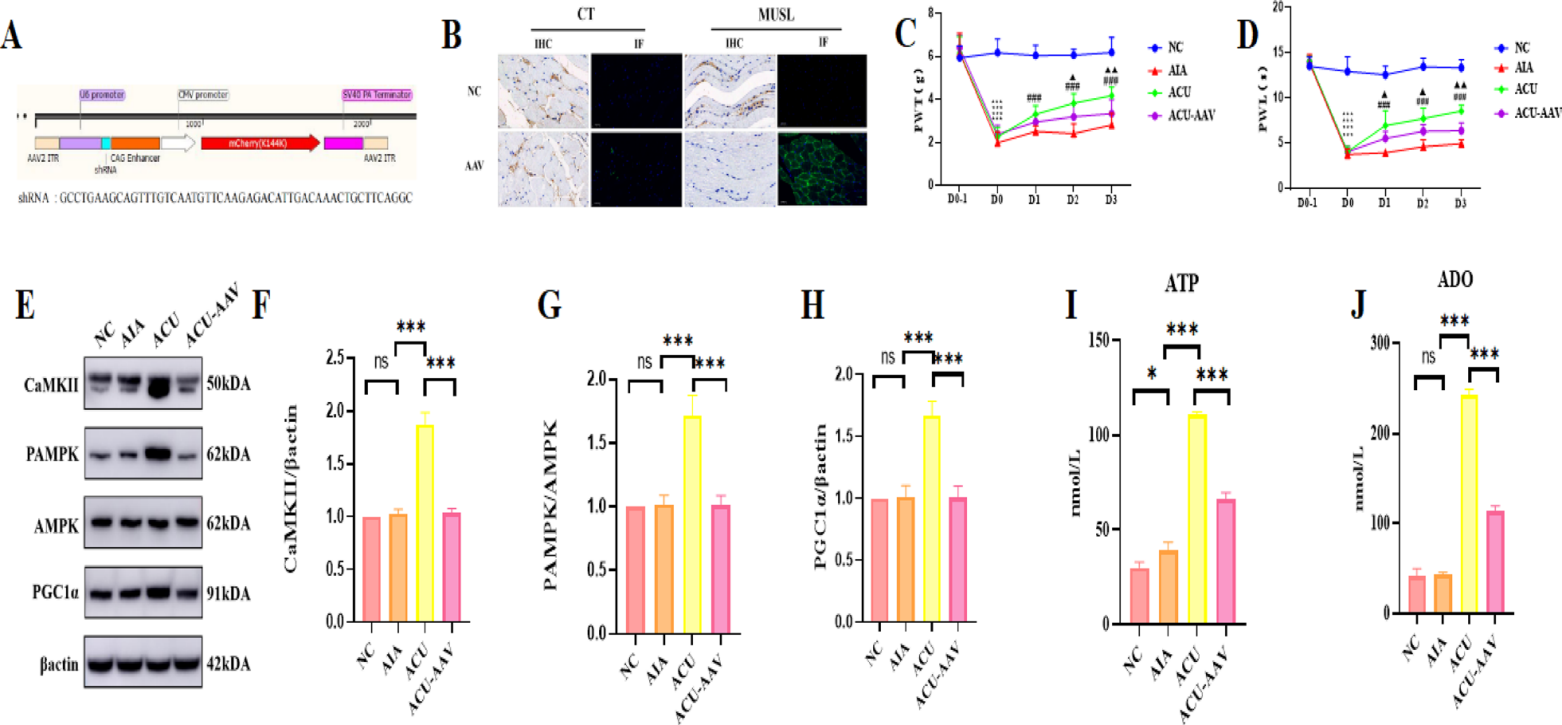
Specific knockdown of TRPV1 in muscle cells can inhibit the CaMKII/AMPK/PGC1α signaling pathway. **A** Construction of an AAV virus for specific knockdown of TRPV1 in muscle cells. **B** Verification of successful virus transfection and TRPV1 knockdown by immunohistochemistry and immunofluorescence. **C**, **D** Effect of injection of the virus on the analgesic effect of acupuncture (PWL and PWT) (n = 6 per group). **E** Western blotting (WB) image of the effect of acupuncture on the protein expression levels of CaMKII, pAMPK, AMPK, and PGC1α after local injection of the AAV virus at the acupoint (n = 6 per group). **F**–**H** Quantitative diagrams of the effect of acupuncture on the protein expression levels of CaMKII, pAMPK, AMPK, and PGC1α after local injection of the AAV virus at the acupoint (n = 6 per group). **I**, **J** Effect of acupuncture on the expression levels of ATP and ADO after local injection of the AAV virus at the acupoint (n = 6 per group). Data are presented as the mean ± SD. *p < 0.05, **p < 0.01, ***p < 0.001

To further validate the central role of TRPV1 on myocytes in acupuncture analgesia, we designed an experimental strategy using adeno-associated virus (AAV). Firstly, we constructed an AAV vector capable of specifically knocking down TRPV1 expression on myocytes (Fig. 8A) and confirmed the successful injection of the virus and its effective knockdown of TRPV1 on myocytes (Fig. 8B). Subsequently, through tests of paw withdrawal latency (PWL) and paw withdrawal threshold (PWT), we found that the pain model was successfully established in the AIA group, and compared with the AIA group, the ACU group showed significant increases in PWL and PWT after acupuncture intervention (p < 0.001), indicating that acupuncture has a significant analgesic effect. Although the PWL and PWT also increased in the ACU-AAV group (the acupuncture treatment group that received AAV injection to reduce TRPV1 expression) (p < 0.05), the magnitude of increase was significantly smaller compared with the ACU group without TRPV1 knockdown (p < 0.01, Fig. 8C and D). This series of results provides strong evidence that TRPV1 on myocytes plays a crucial role in mediating the acupuncture analgesia effect.

**Fig. 9:**
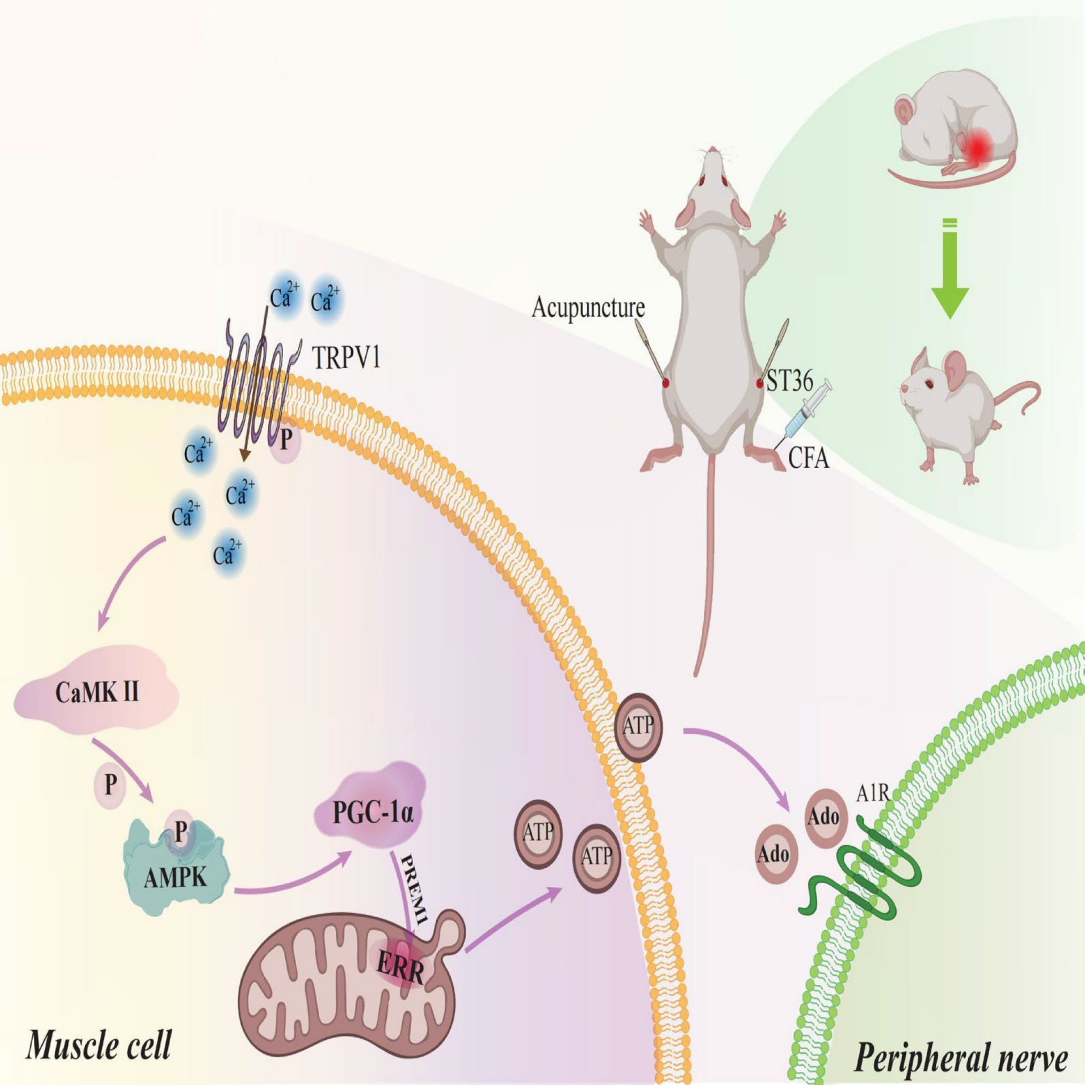
Schematic Diagram Illustrating the Local Effect of Acupuncture in Promoting ATP Metabolism in Muscle Cells at the ST36 Acupoint by Activating the TRPV1/CaMKII/AMPK/PGC1α Signaling Pathway. The Analgesic Effect of Acupuncture on Inflammatory Pain in Mice, with TRPV1 Playing a Central Role in the Mechanism of Acupuncture Analgesia. In particular, the TRPV1/CaMKII/AMPK/PGC1α signaling pathway in muscle cells at the ST36 acupoint significantly promotes ATP metabolism, which is crucial for acupuncture analgesia

Furthermore, to delve deeper into whether CaMKII, pAMPK, AMPK, and PGC1α are downstream components of the TRPV1 pathway, we conducted an experiment by locally injecting adeno-associated virus (AAV, with virus characteristics consistent with previous descriptions) into the acupoint and observed changes in the expression levels of these downstream proteins. WB experimental results showed that the group with local AAV injection at the acupoint effectively inhibited the protein expression levels of CaMKII, pAMPK, AMPK, and PGC1α (p < 0.001, Fig. 8I, J). Concurrently, Elisa experimental results also indicated that this group significantly reduced the expression levels of ATP and ADO (p < 0.001, Fig. 8I, H). These results strongly suggest that these proteins may be located in the downstream pathway of TRPV1 (see Fig. 9).

## Discussion

Acupuncture has been recognized to treat chronic pain by acupuncture at specific parts of the patient’s body (acupoint area) [25]. Acupuncture analgesia is only manifested when the patient has complex feelings of acupuncture (soreness, numbness, heaviness and swelling) after acupuncture operation [26]. After acupuncture, it will cause physical and biological changes in the acupoint area, such as local tissue deformation, displacement, connective tissue winding, muscle fiber fracture, acupoint resistance reduction, etc.; it can also cause chemical and biological changes in the acupoint microenvironment, such as neurosensory excitation, cell function activation, release of related chemicals, etc. Clinically, there are two commonly used acupuncture methods: manual acupuncture (MA) and electroacupuncture (EA) [27]. The MA is to insert the acupuncture needle into the acupoint, and then twist the needle with the hand. In MA, all types of afferent fibers (Abeta, Adelta and C) are activated. In EA, the stimulating current is delivered to the acupoint by an inserted needle [26]. Strong currents sufficient to stimulate Abeta fibers and some Adelta fibers can induce analgesic effects [26]. A large number of studies have shown that a series of cascade reactions occur after acupuncture, including the release of neurotransmitters, the release of endogenous opioids, and the activation of c-fos in the central nervous system [28]. Many brain regions that make up complex networks are involved in acupuncture analgesia. However, there are relatively few studies on the coupling of physical biological changes and chemical biological changes after acupuncture at acupoints and the initiation mechanism of acupuncture at acupoints, and the mechanism of acupuncture analgesia cannot be fully explained. We employed an AIA mouse model to study inflammatory pain, mainly studied the changes of microenvironment in the acupoint area after acupuncture at ST36, aiming to clarify the scientific principle of acupuncture onset.

In the early stage, our team established an experimental model of inflammatory pain in AIA mice (literature) and applied it to the study of the principle of acupuncture initiation. We further screened the most significant intervention time of acupuncture analgesia for 3 days by acupuncture for 1 day, 3 days and 7 days. Therefore, the experiment selected acupuncture for 3 days to study the changes of local microenvironment of acupoints after acupuncture. Mechanically sensitive ion channels mainly include TRP family, Piezo channel and so on. Transient receptor potential (TRP) channels are a class of cation channels located on the cell membrane, which play an important role in sensory neurons. Recent studies have shown that TRP channels are closely related to the occurrence of pain and may become a new target for the treatment of pain. TRPV1 is the most thoroughly studied TRP family ion channel. It is a polyregulated receptor that can be activated by a variety of stimuli, and the downstream pathways guided by different stimuli are different [29]. As a pain sensor, TRPV1 mainly mediates inflammatory pain, neuropathic pain and visceral pain [1]. Inflammatory factors hydrolyze phosphatidylinositol by activating protein kinase C to produce inositol triphosphate and diglyceride, which opens the TRPV1 [30] channel and influxes calcium ions [31]. Injection of TRPV1 antagonists can effectively relieve pain [18, 32]. TRPV2 is essential for phagocytosis, migration and cytokine (TNFα, IL-6) production of mouse macrophages. Targeting TRPV2 may play a beneficial role in diseases in which macrophages and mast cells play a key role. TRPV4 is a non-selective cation channel with moderate permeability to Ca^2+^, which can be activated by moderate temperature (25–34 °C), endogenous inflammatory metabolites (such as arachidonic acid metabolites) and exogenous compounds, and expressed at a very high level in mouse and human skin. TRPV4 is not a heat receptor compared to TRPV1 and TRPV2. The Piezo family is another mechanosensitive ion channel that is considered to be related to acupuncture analgesia. Piezo1 opens its cation channel, allowing Ca^2+^ influx, driving downstream signaling pathways and regulating various physiological processes of cells [33]. Piezo2 mainly mediates mechanically activated currents in fast-adapted sensory neurons and is essential for the response to light touch stimulation. Hu Hongzhen’s research group [34] found that Piezo2 channel is involved in visceral pain hypersensitivity caused by IBS and intestinal obstruction. Mingli Duan found that [35] targeted inhibition of Piezo2 channel may be an effective analgesic method to reduce mechanical hypersensitivity. In this study, five different types of mechanosensitive ion channels with analgesic effects reported in the above literature were screened. The results showed that the expression of TRPV1 and Piezo1 increased after acupuncture, and the increase of TRPV1 was the most significant. Therefore, we mainly studied how TRPV1 was activated and mediated acupuncture analgesia after acupuncture.

The guiding principle for the depth of acupuncture is that the depth of acupuncture should be determined according to the location of the lesion. Different acupuncture depths stimulate the receptors at the acupoints, and the corresponding stimulation effects will also be different. Based on modern anatomy, the depth of needle insertion at acupoints is different, which can act on different anatomical structures and tissue levels. It is generally divided into shallow layer (skin and dermis, fascia layer connective tissue) and deep layer (muscle layer) [36]. Studies have confirmed that the depth of acupuncture needle insertion is closely associated with the analgesic efficacy of acupuncture. Under the same acupoint, different depths of needle insertion also produce different analgesic effects [37]. Therefore, we studied the level of acupuncture analgesia mediated by ST36 acupoint area. After acupuncture of the deep muscle layer, the mice’s perception of mechanical pain decreased, and the expression levels of TRPV1 and pTRPV1 were significantly up-regulated. It is suggested that acupuncture of the deep muscle layer can more effectively promote the expression and activity of TRPV1 than the superficial fascia layer, which is consistent with Professor Ma Qiufu’s research results published in *Nature* [7]. In addition, through the local injection of muscle relaxant in the acupoint area, it was found that acupuncture at the deep muscle layer could exert the analgesic effect of acupuncture and significantly enhance the expression and activity of TRPV1, while the intervention of muscle relaxant could reverse this process, which further confirmed the core role of ST36 muscle layer TRPV1 in the analgesic mechanism of acupuncture.

The cellular localization of TRPV1 varies depending on the cell type and physiological environment. It is mostly expressed in the sarcoplasmic reticulum (SR) and mitochondrial outer membrane of cardiomyocytes and skeletal muscle cells, as well as the endoplasmic reticulum (ER) of DRG neurons [38]. Its localization may be related to its function, such as in pain perception and temperature regulation. In order to further explore the cell types of local acupuncture analgesia after acupuncture, we performed immunofluorescence co-localization experiments on TRPV1 and fibroblasts, macrophages, mast cells and muscle cells. It is worth noting that the number of TRPV1-positive muscle cells also increased significantly, suggesting that muscle cells may be the key cell type of TRPV1-mediated acupuncture analgesia. The enrichment of muscle cells and the arrangement of muscle fibers constitute the basis of acupoints as the reaction site of acupuncture and moxibustion. Acupuncture can cause muscle fiber deformation and activate the TRPV1 channel on the muscle cell membrane, thereby stimulating muscle cells to release bioactive substances and activating nerve receptors to produce analgesic effects.

Studies have found that TRPV1 channel activity is coupled with intracellular inflammatory response-related cell signaling cascades [39]. The functional regulation mechanism of post-translational modifications (PTMs) of TRPV1 is closely related to pain sensitization caused by peripheral inflammatory response. PTMs can promote or inhibit the interaction between molecules through conformational changes, thereby controlling the functional activity of the protein [40]. The phosphorylation and SUMOylation levels of TRPV1 were significantly increased in DRG of inflammatory pain sensitization model mice [41]. Inflammatory mediators can further enhance the function of TRPV1 by activating intracellular signaling cascades [42]. In order to determine the downstream pathways affected by TRPV1, we used a combination of proteomics and phosphoproteomics to further screen for differentially expressed proteins in local acupoints after acupuncture. It was found that these differentially expressed proteins were related to energy metabolism pathways. TRPV1 is mostly expressed on the outer membrane of mitochondria, which is the energy factory of cells. Therefore, the activation of TRPV1 may affect the changes of downstream energy metabolism pathways. Kenneth Maiese found that [43] TRPV1 receptor is not entirely dependent on calcium signaling to affect cell biology, but also closely related to the mechanism targets of mTOR, AMPK and Akt, which play a role in pain sensitivity, stem cell development, cell survival and cell metabolism. The CaMKII/AMPK/PGC1α [44, 45] signaling axis was found to be related to mitochondrial biogenesis. After further verification, we found that acupuncture can activate the local acupoint signaling pathway TRPV1/CaMKII/AMPK/PGC1α, which in turn exerts the analgesic effect of acupuncture.

## Conclusion

This study systematically explored the analgesic effect of acupuncture on inflammatory pain in mice and its mechanism. The study found that acupuncture can significantly improve the thermal pain threshold and mechanical pain threshold in mice, showing a significant analgesic effect. Further analysis revealed that this analgesic effect was closely related to the up-regulation of local TRPV1 expression at ST36. Through gene knockout and antagonist injection strategies, we confirmed the key role of TRPV1 in acupuncture analgesia, and its deletion or functional inhibition will significantly weaken the analgesic effect of acupuncture. In addition, we also found that acupuncture at the deep muscle layer can more effectively promote the expression and activity of TRPV1 than the shallow fascia layer, thereby exerting a stronger analgesic effect. Immunofluorescence double labeling technique further confirmed that muscle cells are the key cell types of TRPV1-mediated acupuncture analgesia. Finally, through the combined analysis of proteomics and phosphorylated proteomics, we analyzed the acupuncture-activated acupoint local signaling pathway, and found and verified multiple key proteins and phosphorylation modification sites related to acupuncture analgesia. In summary, this study not only confirmed the analgesic effect of acupuncture on inflammatory pain in mice, but also revealed the core role of TRPV1 in the analgesic mechanism of acupuncture, especially the important contribution of the expression and activity of TRPV1 in the muscle layer of ST36 to the analgesic effect of acupuncture, which provided a new scientific basis and potential therapeutic target for acupuncture treatment of inflammatory pain.

## Data Availability

The datasets generated and/or analysed during the current study are not publicly available due [REASON WHY DATA ARE NOT PUBLIC but are available from the corresponding author on reasonable request.

## Abbreviations

AAV: adeno-associated virus
ACU: acupuncture
ADO: adenosine
AIA: arthritis-induced model
AMPK: AMP-activated protein kinase
CaMKII: calcium/calmodulin-dependent protein kinase II
CFA: complete Freund's adjuvant
dia-PASEF: data-independent parallel accumulation serial fragmentation
dorsal root ganglion: dorsal root ganglion
DTT: dithiothreitol
EA: electroacupuncture
ECL: enhanced chemiluminescence
Elisa: Enzyme-Linked Immunosorbent Assay
ER: endoplasmic reticulum
IAA: iodoacetamide
KEGG: Kyoto Encyclopedia of Genes and Genomes
KO: knockout
LC-MS: liquid chromatography-mass spectrometry
LSD: least significant difference
MA: manual acupuncture
NC: normal control
PCA: principal component analysis
PCR: Polymerase chain reaction
PTRPV1: phosphorylated TRPV1
PTMs: post-translational modifications
PWL: paw withdrawal latency
PWT: paw withdrawal threshold
ROIs: regions of interest
SC: spinal cord
SEMs: standard errors of the means
SPF: specific pathogen free
SR: sarcoplasmic reticulum
ST36: Zusanli
TCA: trichloroacetic acid
TRP: Transient receptor potential
TRPV1: Transient Receptor Potential Vanilloid 1
WB: Western blotting
WT: wild type
